# Cluster randomised trial on the effectiveness of a computerised prompt to refer (back) patients with type 2 diabetes

**DOI:** 10.1371/journal.pone.0207653

**Published:** 2018-12-05

**Authors:** Maaike C. M. Ronda, Lioe-Ting Dijkhorst-Oei, Rimke C. Vos, Paul Westers, Guy E. H. M. Rutten

**Affiliations:** 1 Julius Centre for Health Sciences and Primary Care, University Medical Centre, Utrecht, The Netherlands; 2 Department of Internal Medicine, Meander Medical Centre, Amersfoort, The Netherlands; 3 Department of Public Health and Primary Care/LUMC-Campus the Hague, Leiden University Medical Centre, The Hague, The Netherlands; Public Library of Science, UNITED KINGDOM

## Abstract

**Aims:**

Information and communications technology (ICT) could support care organisations to cope with the increasing number of patients with diabetes mellitus. We aimed to aid diabetes care providers in allocating patients to the preferred treatment setting (hospital outpatient clinic or primary care practice), by using the Electronic Medical Record (EMR).

**Methods:**

A cluster randomised controlled trial. Physicians in primary and secondary care practices of the intervention group received an advisory message in the EMR during diabetes consultations if patients were treated in the ‘incorrect’ setting according to national management guidelines. Primary outcome: the proportion of patients that shifted to the correct treatment setting at one year follow-up.

**Results:**

47 (38 primary care and 9 internist) practices and 2778 patients were included. At baseline, 1197 (43.1%) patients were in the correct treatment setting (intervention 599; control 598). Advice most often (68.4%) regarded a consultation with the internist. After one year 12.4% of the patients in the intervention and 10.6% in the control group (p = 0.30) had shifted to the correct setting. Main reasons for not following advice were: 1. physician’s preference to consider other treatment options; 2. patients’ preferences.

**Conclusions:**

We could not find evidence that using the EMR to send consultation-linked advice to physicians resulted in a shift in patients. Physicians will not follow the advice, at least partly due to patients’ preferences.

## Introduction

Patients with diabetes require regular check-ups by physicians and nurses. Diabetes management needs to become as efficient and cost-effective as possible to deal with the increasing number of patients with diabetes. In the Netherlands about 85% of patients with type 2 diabetes mellitus are treated by general practitioners collaborating with practice nurses in a primary care setting [1] according to national clinical guidelines for primary care [2]. Only patients that are in need of more complex care are referred to a hospital based internist or endocrinologist, collaborating with specialised diabetes nurses. There is national agreement between primary and secondary care with regard to the targets of diabetes care and the setting in which diabetes care should take place [3], to which we will refer as management guidelines.

Almost all general practitioners are organised in care groups that assume financial and clinical accountability and in turn subcontract individual care providers (physicians, dieticians, podiatrists) [1, 4]. For many reasons the costs per patient in secondary care are higher than in primary care. Both because of quality of care and of cost-effectiveness, correct identification of patients who might benefit from referral to an internist and identifying patients that can be treated in primary care is relevant. In a recent study in Denmark, patients remained in specialist care much longer than guidelines stipulated [5]. Further, in patients with good cardiometabolic control a six-monthly instead of three-monthly monitoring does not compromise outcome and is cost-saving [6]. A patient portal that provides patients access to their own medical record and with an option of secure electronic communication with the provider can be used as a substitute of an office visit once or twice a year [7].

We hypothesise that targeted use of information technology by an alert according to the national management guidelines in the patient’s electronic medical record (EMR) will result in better treatment allocation of patients with diabetes. Therefore, it was aimed to investigate the effectiveness of such messages provided to physicians and to increase our understanding of the reasons of not adhering to advice.

## Materials and methods

### Design and setting

This cluster randomised controlled trial was performed between October 2013 and October 2014 in **‘**Diamuraal’, a care group of 66 primary care practices and an outpatient clinic with the practices of 10 internists. It provides diabetes care to over 12.000 patients with type 1 and type 2 diabetes mellitus. All health care providers work with the same EMR, with only one physician (primary care physician or internist) designated as main physician. He or she can use the message function of the EMR to consult another physician who then has temporary access (change of treating physician) to the medical information of a patient. All patients can request a login to a patient web portal that gives them access to their entire personal EMR, including clinical notes, physical examination, laboratory results and secured electronic message with their provider (www.digitaallogboek.nl) [7].

All physicians were invited to participate in this study. Practices were only included if all physicians consented to participate. Their patients with type 2 diabetes received an information letter about the trial, stating that after informed consent the final decision to follow advice or not should be a shared decision of patient and treating physician.

The study was approved by the Medical Ethics Committee of the University of Utrecht (protocol number 13-039/C; February 13^th^ 2013). Practices were recruited between March 1^st^ and May 30^th^ 2013, patients were included between April 1^st^ and August 1^st^ 2013. Data were collected from the central database at start (October 9^th^ 2013) and end of study (October 9^th^ 2014). At the end of study period patients received a questionnaire, which was sent between October 1^st^ and October 30^th^ 2014. The study was registered at Clinicaltrials.gov (NCT02229110, August 29^th^ 2014). There was a delay in registration due to the maternity leave of the first author and miscommunication with the co-authors. As a result the protocol was registered after start of the patient recruitment. The authors confirm that they are not performing any trial related to this intervention.

### Randomisation

Primary care practices were randomised with stratification of 1. Practice size (small or large, with a cut-off point of 150 patients with type 2 diabetes); 2. Practice type (group or single handed practice) and 3. Practice location (city or rural). The 10 internists were randomised separately, stratification for number of diabetes patients of whom they are the treating physician (cut-off point of 100 patients). Randomisation was executed at the research centre via a computer generated sequence by an independent researcher.

### Assessment of the setting

All patients were assessed whether they were treated in the right setting according to the management guidelines for primary and secondary care on treatment setting [3]. For example: in a 68 years old patient with an estimated glomerular filtration rate (eGFR) value of 40 ml/min, the primary care practice should plan an electronic consultation with the internist, and a patient with the same age and an eGFR of 29 ml/min should be referred.

In order to assess the correctness of the treatment setting, we created an algorithm (S1 Table) based on management guideline cut-off values. Some targets in the guideline are subjective; if possible these were objectified by a team consisting of a general practitioner, a specialised diabetes nurse and an internist. At the end of the study all patients were assessed again, blinded for randomisation allocation.

### Intervention

If a patient in the intervention group was not treated correctly according to the algorithm a message was provided with advice to change setting. The message was sent to the EMR email box of the treating physician and also presented as a pop up in the monitor screen upon opening it, accentuated in yellow. Besides advice to change the treatment setting of the patient, the message gave an explanation on which marker(s) it was based (S1 Table). The health care provider was expected to discuss this advice with the patient and to decide to follow it or not. In case it was overruled, the care provider was asked to document the reason for it (S2 Table). Because either the nurse or the physician sees the patient about four times a year, they were in the position to discuss the advice several times during the study period. The advice was sent at the start of the study and again after six months to physicians who had not yet responded. Patients with access to their EMR also received the message and they were encouraged to discuss it with their provider. No message was sent to providers and patients in the intervention group who were treated in the right setting, according to the algorithm.

A general practitioner could receive 3 different types of advice, namely 1. consult the internist using the EMR; 2. refer the patient to the internist; and 3. instruct the patient to use the patient portal for self-monitoring instead of office visits. The internist could only receive one type of advice: referral back to the primary care physician.

Advice could be based on one or more markers. Even in case of several markers, only one advice type was sent. For example: we could send the physician a message with advice for consultation with an internist because this patient had blood pressure above target or send a similar message because the patient had a combination of high blood pressure *and* an abnormal lipid profile.

Furthermore, there were patients that had one or more markers leading to advice for consultation and other markers that lead to advice for referral. In these patients both advices were sent simultaneously, for example a high HbA1c could warrant the advice for consultation with an internist while at the same time this patient could also have a high triglyceride leading to advice for referral. In such a situation both messages were sent to both the provider and the patient.

### Control group

The patients in the control group received care-as-usual, without any messages sent to their diabetes care provider or to the patients themselves whether or not treated in the right setting.

### Outcome measures

The primary outcome was the proportion of patients that changed to the correct treatment setting after one year. Secondary outcomes were the number of different types of advice and the markers they were based on. Furthermore, we measured the reasons for non-adherence to the advice.

At baseline and after one year the following measures were collected from the central database of Diamuraal: patient’s age; current treatment setting; type of diabetes (diabetes mellitus type 1, type 2, LADA or MODY); fasting glucose; HbA1c; systolic blood pressure (SBP); Body Mass Index (BMI); lipids (total cholesterol, LDL- and HDL-cholesterol, triglyceride and total/HDL-cholesterol ratio); kidney function (eGFR, albumin/creatinine ratio, serum creatinine and albuminuria) and the following complications: diabetic ulcer, amputation, retinopathy, myocardial infarction, angina pectoris, heart failure, stroke, transient ischemic attack, peripheral arterial disease. Also the use of oral diabetes medication, insulin (pump), lipid or blood pressure lowering medication, a platelet inhibitor and anticoagulants was assessed.

In the intervention group data were collected whether the physician followed advice and, if not, the reasons for not following it (predefined options with more than one possible reason to give and room for free text) (S2 Table).

### Statistical analysis

The sample size was calculated on the proportion of patients shifting from an incorrect to a correct setting after one year. We expected that at baseline 25% of patients were at the incorrect setting. After one year this proportion was assumed to be decreased to 12.5% in the intervention group, while in the control group the situation would remain the same. With these assumptions, 2234 patients had to be included to detect a significant difference between groups with 90% power and α of 5%, taking an estimated intra-cluster correlation of 0.075 into account.

Analyses were according to the intention-to-treat principle, with patients lost-to-follow up analysed as ‘no change in setting’. Baseline differences between groups were analysed with independent samples t-test for continuous variables and Chi-square test for categorical data. The change in setting *within* groups after one year was analysed with McNemar’s test. The reasons for different advice, physician’s adherence to the advice and the reasons for non-adherence were described with counts and percentages. To determine an intervention effect generalised linear mixed model was used, adjusted for clustering, treatment location (primary or secondary care) at baseline and baseline setting assessment. Analyses were performed using SPSS version 21 (SPSS Inc. Chicago, IL, USA).

## Results

Of the 66 primary care practices invited, 38 (57.6%) agreed to participate. All 10 internists agreed to participate, but one internist was excluded because he is the main physician of only 17 type 2 diabetes patients. Thus 47 practices were included.

From primary care 6755 patients were invited and 2382 (35.3%) returned the consent form (mean number of returned consent forms per practice 63, range 20–138). From secondary care 1633 patients were invited, 396 (24.2%) returned the consent form (mean number of returned forms per practice 44, range 6–80).

Participating patients and non-participants did not differ in age (68.5±10.8 years and 68.5±13.2 years (p = 0.95), respectively), but significantly more males participated (57.9% versus 47.8% (p<0.001)).

**Fig 1:**
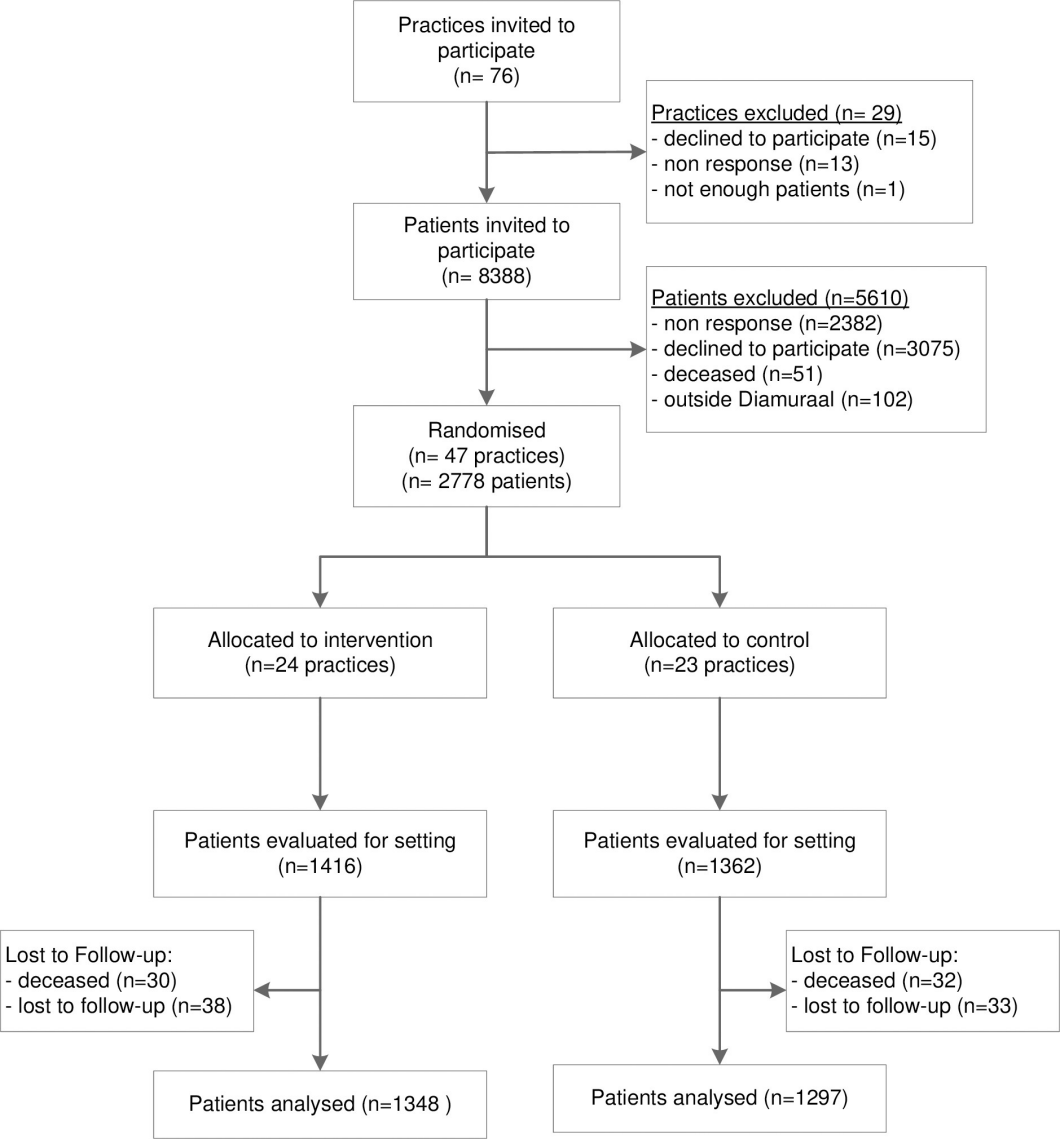
Flowchart.

At one year follow-up complete data from 1348 (95.2%) patients in the intervention group and 1297 (95.2%) patients in the control group were available (Fig 1).

At baseline 599 (42.3%) patients in the intervention group and 598 (43.9%) patients in the control group were treated in the correct setting (Table 1). After one year 175 out of 1416 (12.4%) patients in the intervention group and 144 out of 1362 (10.6%) patients in the control group (p = 0.30) had shifted to the correct setting; 642 (45.3%) patients in the intervention group and 620 (45.5%) in the control group remained in the incorrect setting (p = 0.67). Most patients remained in the setting they started, which was incorrect for most patients in primary care and correct for those in secondary care (Table 2).

**Table 1:** Baseline characteristics of practices and patients.

	Intervention	Control
**Practices (n = 47)**	**24**	**23**
Primary care	20	18
Secondary care	4	5
*Primary care practices*		
Location (city / rural)	12/8	11/7
Practice (group / single handed)	12/8	10/8
Size (≤ 150 patients / >150 patients)	10/10	9/9
*Secondary care practices*		
Size (≤ 100 patients / > 100 patients)	1/3	2/3
**Patients (n = 2778)**	**1416**	**1362**
Age, years	68.3 ± 10.8	68.8 ± 10.8
Gender, male	811 (57.3)	798 (58.6)
HbA1c, % (mmol/mol)	6.8±0.9 (51.3±9.7)	6.9±0.9 (51.7±10.1)
BP systolic, mmHg	134.1±15.6	132.5±15.0
LDL-cholesterol, mmol/l	2.3±0.9	2.3±0.8
*Patients in primary care (number)*	1235	1147
Correct setting	447 (36.2)	422 (36.8)
Incorrect setting	788 (63.8)	725 (63.2)
*Patients in secondary care (number)*	181	215
Correct setting	152 (84.0)	176 (81.9)
Incorrect setting	29 (16.0)	39 (18.1)

Patients: categorical variables are total number (percentage), continuous variables are mean±SD.

**Table 2:** Shift in setting within groups after one year.

	Intervention group (n = 1416)			Control group (n = 1362)		
Baseline	Follow-up			Follow-up		
**Total Group (n = 2778)**	**Correct**	**Incorrect**	**Total**	**Correct**	**Incorrect**	**Total**
Correct	419 (29.6)	180 (12.7)	599	438 (32.2)	160 (11.7)	598
Incorrect	175 (12.4)	642 (45.3)	817	144 (10.6)	620 (45.5)	764
Total	594	822	1416 (100)	582	780	1362 (100)
p-value *	*0*.*83*			*0*.*39*		
**Secondary Care (n = 396)**	**Correct**	**Incorrect**	**Total**	**Correct**	**Incorrect**	**Total**
Correct	123 (68.0)	29 (16.0)	152	151 (70.2)	25 (11.6)	176
Incorrect	11 (6.1)	18 (9.9)	29	21 (9.8)	18 (8.4)	39
Total	134	47	181 (100)	172	42	215 (100)
p-value *	*0*.*01*			*0*.*66*		
**Primary Care (n = 2382)**	**Correct**	**Incorrect**	**Total**	**Correct**	**Incorrect**	**Total**
Correct	296 (24.0)	151 (12.2)	447	287 (25.0)	135 (11.8)	422
Incorrect	164 (13.3)	624 (50.5)	788	123 (10.7)	602 (52.5)	725
Total	460	775	1235 (100)	410	737	1147 (100)
p-value *	*0*.*50*			*0*.*49*		

Data are numbers (percentages).

* McNemar

No intervention effect for change in treatment setting after one year was found (adjusted odds ratio 0.99 (95% CI 0.77–1.28)).

Advice to change treatment setting was applicable to 817 (57.7%) persons in the intervention group with an incorrect setting at baseline (Table 2). In 559 persons, the general practitioner was advised to consult an internist (292 patients with sole advice for consultation and 267 patients with both an advice for consultation and advice for referral), most frequently based on HbA1c values above target (n = 220) or signs of kidney complications (n = 195). In 451 patients, the general practitioner was advised to refer to an internist (184 patients with sole advice for referral and 267 patients in combination with a consultation advice), mainly based on a SBP above target or the presence of a high BMI (Table 3).

**Table 3:** Number and frequency of different markers leading to advice at baseline.

Markers for consultation of an internist (n = 559 patients)	
Diabetes mellitus other than type 2	6 (0.9)
Probability of diabetes other than type 2	5 (0.7)
High HbA1c	220 (31.1)
Known with high systolic blood pressure for a short period	110 (15.5)
Inadequate lipid profile	140 (19.8)
Presence of moderate kidney complications	195 (27.5)
Presence of diabetic ulcer	24 (3.4)
Presence of macroangiopathy	8 (1.1)
**Markers for referral (n = 451 patients)**	
Known with high systolic blood pressure since long time	151 (26.8)
Probability of familial hyperlipidemia	114 (20.2)
High triglyceride level	2 (0.4)
Presence of severe kidney complications	32 (5.7)
Presence of retinopathy	119 (21.1)
Presence of body mass index above 35 kg/m2	145 (25.8)
**Markers for instructing patients for self-monitoring (n = 45 patients)**	
Stable disease with good cardiometabolic control	45 (100)
**Markers for advice for back referral (n = 29 patients)**	
Reaching personal treatment goals in secondary care	29 (100)

Advice for change in treatment setting could be based on one or more markers, for definition of the markers see S1 Table. Data are numbers (percentages).

Advice for consultation of a medical specialist was intentionally followed in only 5.9% of the concerning advices, the advice to refer the patient in only 8.2% and the advice for self-monitoring in 24.4%. In about one in three ((34.5%) cases the internists followed the advice to refer people back to the GP. If GPs did not follow the advice to consult an internist, most frequently they reported not to do so because they wanted to make treatment adjustments themselves. If patients were not referred by the primary care physician, this was hardly (6.7%) the result of a patient’s request. In contrast, internists reported that if they did not refer a patient back, in 40% this was because of patients’ request (Table 4).

**Table 4:** Physician response to advice given and main reasons for not following the advice.

	Consultation	Referral	Self-monitoring	Back referral
Response physician after receiving advice*	**N = 559**	**N = 451**	**N = 45**	**N = 29**
Will follow advice	33 (5.9)	37 (8.2)	11 (24.4)	10 (34.5)
Will not follow advice	390 (69.8)	369 (81.8)	31 (68.9)	18 (62.1)
No response	136 (24.3)	45 (10.0)	3 (6.7)	1 (3.4)
Main reasons for not following advice**	**N = 541**	**N = 492**	**N = 53**	**N = 15**
Physician wants to make treatment adjustments first	182 (33.6)	98 (19.9)		
Doubt about compliance/lifestyle/therapy adherence by patient	56 (10.4)	40 (8.1)		
At patient’s request	57 (10.5)	33 (6.7)	11 (20.8)	6 (40.0)
No retinopathy present *(specific for referral)*		57 (11.6)		
Referral in the past, not documented in the electronic medical record		45 (9.1)		
Patient has no glucose and/or blood pressure monitor at home			10 (18.9)	
Other comorbid conditions / recent complication			11 (20.8)	6 (40.0)

Data are total number (percentage).

* N = number of advice type given

** physicians could provide more than one reason for not following the advice

## Discussion

This study shows that over 50 percent of patients with type 2 diabetes were not treated in the correct setting according to nationally agreed guidelines. This percentage was twice as high as expected. Sending a computerised prompt to raise physicians’ awareness of the situation, combined with advice for the preferred treatment setting did not result in a shift of the patient flow in the desired direction. Most general practitioners did not adhere to the advice, mostly because they preferred adjustments of the therapy first. Also patient preference was an important reason for non-adherence.

Several reasons are known from literature why physicians do not follow clinical practice guidelines, e.g. because they are not aware of them or do not agree with [8–10], or recommendations are controversial, non-specific or not evidence based [11]. In the Netherlands the *diagnostic and therapeutic guidelines* on type 2 diabetes in primary care are developed by the Dutch College of General Practitioners, they are highly evidence-based, firmly embedded in primary care and with a high adherence rate. All general practitioners are considered to have experience to follow these guidelines, which provide a stepwise approach for blood glucose lowering therapy. If an adequate diabetes control is not achieved (for whatever reason), the patient should be referred to secondary care [2, 3]. The *national management guideline* on type 2 diabetes, defining the collaboration between internists / endocrinologists and general practitioners had been published less than 2 years prior to this study and is consensus-based [3]. Both types of guidelines pass an agreement procedure among physicians from the Dutch College of General Practitioners and the Dutch Society of Internal Medicine. Nevertheless, it must be kept in mind that limited evidence is available to support the (cost-) effectiveness of shared care programs for chronic diseases in general and type 2 diabetes in particular [12–14]. Maybe physicians do not agree with some advice, e.g. it seems questionable whether all physicians agree with advice for referral in case of high BMI. We would like to recommend that collaboration agreements and guidelines about collaboration between primary and secondary care undergo an extensive testing in the field. Furthermore, the management guideline is consensus based instead of evidence-based which lowers the compliance with the agreement [11]. We feel it needs a more extensive agreement procedure even before implementation, with testing and feedback from more physicians in order to gain support.

Notably, advice for consultation because of high values of HbA1c was based on at least 2 measurements above target and the prerequisite that this situation had existed for over one year. However, it is possible that general practitioners could have adjusted the diabetes treatment resulting in a better *fasting glucose level*, and this could be a reason for the physician not to follow the advice to consult an internist immediately. Nevertheless, even after having been made aware of the situation, during the follow-up of another whole year, on average 79% of primary care patients (624 out of 788) incorrectly remained treated in solely the primary care setting.

Furthermore, attitudes and preferences from both physicians and patients can be a reason for non-adherence [15–19]. A national survey showed that Dutch general practitioners felt that guideline adherence in general leads to improved patient care and that they have a high perceived adherence to guidelines especially with respect to recommendations for referral [16]. However, during a face-to-face consultation with an individual patient, there are reasons for non-adherence. Physicians may feel that guidelines are no more than suggestions and do not fit individual patients [20]. This might also be true for the physicians in our study, as we found that the main reasons for not following the advice were the physician’s wish to make treatment adjustments first as well as patients’ preferences to remain in the current treatment setting. These preferences could be the results of a long-term relationship in which they have built trust upon each other, and therefore hesitate to change setting. Another aspect may be their view on cost aspects. First of all, in the Dutch health care system, primary care (GP) appointments are completely covered by the national health insurance system, whereas patients have a personal liability scheme on medication and secondary care treatment. By denying secondary care referral and choosing for basic, cheap medication, patients can save costs. Cost aspects may also drive the general practitioner to try and prevent referral, to save on the national health budget for secondary diabetes care. However, in our opinion an alternative explanation is likely more relevant, namely that primary care nurses and physicians were confident in their ability to achieve the same results as in secondary care by adjusting treatment regimens, as they had learned from a long time of intense collaboration with the internal medicine specialists. Should this be true, then referral guidelines should be loosened, e.g. advisory prompts for consultation less strict and advisory prompts for referral replaced more widely by prompts for consultation. Whether such self-confidence is justified, may become clear from studying treatment outcomes in the intervention versus the control group. Finally, the fact that patient’s preferences also accounted for a small percentage of the reasons not to refer or refer back, implies that an EMR should contain smart digital information on patient preferences. Patient preference is an often reported reason for guideline non-adherence, which might be valid and not compromising quality of care [18]. The adherence of the internists to our advice to refer back patients to primary care after targets are met or in case of stable disease might reassure primary care physicians and their patients that intensifying treatment setting could indeed be temporarily.

Strength of the current study is a large population of patients with type 2 diabetes both from primary and secondary care physicians. To the best of our knowledge this is the first study on the effect of advice to health care providers by using the EMR to change treatment setting. However, there are also limitations. Our interpretation of guideline items that were imprecisely formulated, in order to run the algorithm, could have led to more patients in the wrong treatment setting at baseline, although it was done and agreed upon by a team of different diabetes care providers. In daily practice physicians may interpret “persistent high level” loosely and this could lead to clinical inertia. As a result the patient is likely to be worse off. With respect to physician characteristics, although almost 60% of the general practices an almost all internists participated we cannot rule out selection bias. We cannot rule out that physicians who participated are more interested in diabetes than those who did not. Assuming that they are more interested, they might have been more confident in their ability to adjust treatment, without the need for consultation or referral. In this way, selection bias would result in a higher non-adherence to the advice message. Furthermore, literature shows that female physicians prefer to prescribe different types of antihypertensive medication to patients with type 2 diabetes with hypertension compared to male physicians [21]. Overall reasons for referral to secondary care are different for female physicians [22]. We do not have any information about age and gender of the physicians and nurse practitioners, so we are unable to explore the impact of this on our findings. With regard to patient characteristics, a second selection round took place when patients were invited, with 35.3% (primary care) and 24.2% (secondary care) participants. There was a difference in gender but not in age with more males in the participating group. A previous study showed that the odds of referral in a general practice increase with age and especially with the presence of morbidity, but that the effect of gender was very small and most of the variation in referrals remain unexplained [23]. In that study there were slightly more females referred compared to males. In our study there was an overrepresentation of males. It is possible that this could have led to less referrals in our study due to males seemingly being referred less compared to females but as the effect of age and gender combined only explain 5% of the variation in referrals we feel that this did not affected our results. In the Netherlands a patient needs a referral in order to consult an intern medicine specialist. This might be considered a limitation with regard to the generalisability of the results of this study. However, in our opinion also for health care systems with direct access to medical specialists our study is relevant both for physicians as well as for policy makers. Both general practitioners and specialists worldwide are working with EMR based systems are able to incorporate a computerised message, prompt or pop-up to remind the physician to adjust treatment. Our study shows that their effectiveness will depend on human decisions during consultation and local collaboration agreement.

In conclusion, we could not find evidence that a consultation-linked electronic advice to physicians that was based upon nationally agreed guidelines to consult an internal medicine specialist or to refer the patient with type 2 diabetes (GP) or to refer patients back (specialist) resulted in a shift of patients. Both patient and physician related factors play a role in not following the advice. The content of the guidelines may be discussed.

## Supporting information

S1 Table Four different types of advice and algorithm on which the advice is based. (DOC)

S2 Table Reasons for not following the advice. * Predefined reasons at baseline; † Reasons added after evaluating free text; ‡ We checked all these responses: in every case the physician provided this as an answer he was neglecting the specific content of the management guidelines at this point; § i.e. added value of the internist in case of already long term treatment by surgeon (diabetes ulcer), ophthalmologist (diabetes retinopathy) and cardiologist (wrongfully believes that cardiologist takes care of diabetes treatment), or in case of options of discussion new/different medication when there were previous side-effects.
(DOC)

S1 File CONSORT checklist. (DOC)

S2 File Protocol submitted to ethics committee. Protocol submitted to Medical Ethics Committee of the University of Utrecht for assessment if participants are subjected to the Medical Research Involving Human Subject Act (WMO). In Dutch.
(DOCX)

S3 File Protocol in English (only main form). (DOCX)
