# Supplementary material for: Cluster randomised trial on the effectiveness of a computerised prompt to refer (back) patients with type 2 diabetes
Source: PLoS One. 2018 Dec 5;13(12):e0207653. doi: 10.1371/journal.pone.0207653 (PMC6281259; doi:10.1371/journal.pone.0207653)
Supplement: S1 Table — (DOC) [file pone.0207653.s001.doc]

| **Advice 1**  based on: | **This patient should receive a consultation by an internist using the EMR** |
| --- | --- |
| **Diabetes Mellitus other than type 2** | One of the following:   - Diabetes type 1 - Latent Auto-immune Diabetes in Adults (LADA) - Maturity-Onset Diabetes of the Young (MODY) |
| **Probability of diabetes other than type 2** | One of the following:   - Age <25 years - The first glucose value >25 mmol/l in combination with a first BMI <27 kg/cm2 |
| **High HbA1c**  (in mmol/mol) | Duration of diabetes mellitus more than 1 year and one of the following (at least two values above target and for more than one year):   - HbA1c > 53 and no medication - HbA1c > 53 and age < 70 years - HbA1c > 58 and age ≥ 70 years and duration of diabetes less than 10 years - HbA1c > 64 and age ≥ 70 years and duration of diabetes more than 10 years   Duration of diabetes mellitus between 6 months to 1 year and one of the following:   - HbA1c > 58 and age < 70 years - HbA1c > 64 and age ≥ 70 years - HbA1c > 69 |
| **High systolic blood pressure** | - If age <80 years: the last two values were > 140mmHg (within the last 6 months) - If age ≥80 years: the last two values were > 160mmHg (within the last 6 months) |
| **High Lipid profile** | One of the following:   - LDL-cholesterol > 2.5 mmol/l (for at least one year) and SCORE ≥ 20% - Triglyceride > 6 mmol/l (within the last six months) and   SCORE = Systematic COronary Risk Evaluation (10 year risk of fatal CVD in Europe) |
| **Presence of kidney complications** | One or more of the following (the most recent value):   - If age ≥65 years: eGFR value between 30 and 45 ml/min - If age <65 years: eGFR value between 45 and 60 ml/min - If male: albumin/creatinine ratio between 2.5 and 25 mg/mmol - If female: albumin/creatinine ratio between 3.5 and 35 mg/mmol - Most recent blood creatinine value is at least 25% higher than the second to last |
| **Presence of diabetic ulcer** | Presence of diabetes ulcer |
| **Presence of macroangiopathy** | One or more of the following within the last year:   - Myocardial infarction - Coronary artery disease - Angina pectoris - Heart failure - Cerebrovascular accident - Transient ischemic accident - Peripheral artery disease |
| **Advice 2**  based on: | **This patient should be referred to secondary care** |
| **High systolic blood pressure** | - If age <80 years: the last two values were > 140mmHg (for more than 6 months) - If age ≥80 years: the last two values were > 160mmHg (for more than 6 months) |
| **Probability of familial hyperlipidemia** | One of the following (one measure once):   - Total cholesterol of 8 mmol/l or higher - Total cholesterol/LDL-cholesterol ratio of 8 or higher - LDL-cholesterol of 5 mmol/l or higher |
| **High triglyceride** | Triglyceride 6 mmol/l or higher for at least 6 months |
| **Presence of kidney complications** | One or more of the following (the most recent value):   - If age ≥65 years: eGFR value below 30 ml/min - If age <65 years: eGFR value below 45 ml/min - If age <40 years: eGFR value below 90 ml/min - Albuminuria ≥200 mg/ml - If male: albumin/creatinin ratio ≥25 g/mol - If female: albumin/creatinin ratio ≥35 g/mol |
| **Presence of retinopathy** | - Presence of retinopathy |
| **Presence of high Body Mass Index** | - Value ≥ 35 kg/m2 |
| **Advice 3** | **This patient should substitute one or more clinic visits by self-monitoring using the patient portal** |
| **Stable disease with good cardiometabolic control** | All of the following:   - HbA1c ≤ 53 mmol/mol - Systolic blood pressure   - If age < 80 years: value below 140 mmHg   - If age ≥ 80 years: value below 160 mmHg - LDL-cholesterol ≤ 2.5 mmol/l - Total cholesterol ≤ 4.5 mmol/l - Total cholesterol / HDL-ratio < 8 - Kidney function:   - If age ≥65 years: eGFR value above 40 ml/min   - If age 40-65 years: eGFR value above 60 ml/min   - If age < 40 year: eGFR value above 90 ml/min - Body Mass index < 35 kg/cm2 - In the last 2 years no new:   - Myocardial infarction   - Coronary artery disease   - Angina pectoris   - Heart failure   - Cerebrovascular accident   - Transient ischemic accident   - Peripheral artery disease   - Retinopathy   - Diabetes ulcer   - Amputation |
| **Advice 4** | **This patient should be referred back to the primary care** |
| **Reaching personal treatment goals** | All of the following:   - No insulin pump - Medication classes have not been changed the last six months (concerning oral diabetes medication, insulin, lipid lowering medication, blood pressure lowering medication and blood thinning agent) - No new dietitian advice in the last six months - In the last 12 months no proteinuria or macroalbuminuria defined as:   - Albumin in urine ≥ 200 mg/ml   - If male: albumin/creatinine ratio ≥25 mg/mmol   - If female: albumin/creatinine ratio ≥35 mg/mmol - In the previous years no new:   - Myocardial infarction   - Coronary artery disease   - Angina pectoris   - Heart failure   - Cerebrovascular accident   - Transient ischemic accident   - Peripheral artery disease   - Retinopathy   - Diabetes ulcer   - Amputation   Furthermore, if treatment in secondary care has only recently started, between 3 and 12 months before, all of the following:   - Good glycemic control (in mmol/mol):   - HbA1c ≤ 53 and age < 70 years   - HbA1c ≤ 58 and age ≥ 70 years and duration of diabetes less than 10 years   - HbA1c ≤ 64 and age ≥ 70 years and duration of diabetes more than 10 years - Good systolic blood pressure:   - If age < 80 years: value below 140 mmHg   - If age ≥ 80 years: value below 160 mmHg - LDL-cholesterol ≤ 2.5 mmol/l - Total cholesterol ≤ 4.5 mmol/l - Total cholesterol / HDL-ratio ≤ 3.5 - Triglyceride value < 2 mmol/l - eGFR > 45 ml/min   Or, if treatment in secondary care has already been lasting more than 12 months, all of the following:   - Kidney function stable:   - If age < 65 years: eGFR above 45 ml/min for all values in the last six months   - If age ≥ 65 years: eGFR above 30 ml/min for all values in the last six months - Improvement of most recent HbA1c value < 11 mmol/mol compared to the second to last value - Systolic blood pressure below 180 mmHg for the last 2 recent values |
