# Supplementary material for: Cluster randomised trial on the effectiveness of a computerised prompt to refer (back) patients with type 2 diabetes
Source: PLoS One. 2018 Dec 5;13(12):e0207653. doi: 10.1371/journal.pone.0207653 (PMC6281259; doi:10.1371/journal.pone.0207653)
Supplement: S2 Table — * Predefined reasons at baseline; † Reasons added after evaluating free text; ‡ We checked all these responses: in every case the physician provided this as an answer he was neglecting the specific content of the management guidelines at this point; § i.e. added value of the internist in case of already long term treatment by surgeon (diabetes ulcer), ophthalmologist (diabetes retinopathy) and cardiologist (wrongfully believes that cardiologist takes care of diabetes treatment), or in case of options of discussion new/different medication when there were previous side-effects. (DOC) [file pone.0207653.s002.doc]

| **General reasons (applicable in response of all four advice messages)** | |
| --- | --- |
| *  *  *  * | At patient’s request  Life expectancy of this patient is less than one year  There is reasonable doubt about compliance, lifestyle and adherence to therapy  After consideration there were missing values in the EMR and we have added the missing values, this advice should probably not reappear again |
| **Reasons applicable in advice for consultation and in advice for referral** | |
| *  *  *  †  †  †  †  †  †  †  †  † | The situation has already been discussed with the internist and no further improvement is expected  This patient is already evaluated by the internist for this complication without using the EMR of Diamuraal  The current treatment team has room for treatment adjustments  This patient is treated by the cardiologist (in case of blood pressure, high lipids or macroangiopathy)  There is another comorbid condition  Due to age or social situation  I disagree with the advice ^‡^  There is no high blood pressure but there is white coat hypertension  I doubt that consulting an internist has added value ^§^  We have already started taking steps in agreement with your advice  The patient doesn’t want a change in medication  We believe that this is not needed with this patient (values are marginaly high or stable high) |
| **Reasons applicable only in advice for consultation** | |
| *  *  *  * | In hindsight this is not a diabetes type 1, LADA or MODY but indeed a diabetes mellitus type 2  There is a temporary metabolic complication due to infection / surgery / prednisone  There is alcohol abuses  The ulcer is superficial, existing less than 2 weeks and there are no signs of peripheral artery disease |
| **Reasons applicable only in advice for referral** | |
| *  * | There is no progressive retinopathy  BMI is above 35 but primary care treatment is still continuing |
| **Reasons applicable only in advice for self-monitoring** | |
| *  *  *  *  *  †  †  †  †  † | Medication has been adjusted within the last 8 weeks  This patient does not have a glucose and/or blood pressure monitor at home  This patient is not interested in access to his personal health record (patient portal)  This patient does not have access to the internet  This patient is not able to understand his personal health record (patient portal)  Glucose treatment still needs adjustments (newly diagnosed or hypoglycemic episodes)  Other comorbid disease or expected problems that make self-monitoring undesirable  Due to age or social situation  This practice doesn’t have the resources for self-control by patients  I believe this patient is not suited for self-control (unspecified) |
| **Reasons applicable only in advice for referral back** | |
| *****  *****  *****  *****  *****  *****  *****  *****  ***** | This patient is using or will be using an insulin pump  Medication is recently adjusted, the effect needs to be evaluated first  There is a severe comorbid condition  There is severe or progressive micro-albuminuria or decline in kidney function  This patient receives dialysis  This patient has a recent (in the last 12 months) complication  This patient is currently under counselling by a psychologist  The primary care practice of this patient doesn’t provide intensive insulin treatment  The primary care practice of this patient doesn’t accept the referral back of this patient |
