# Supplementary material for: Cluster randomised trial on the effectiveness of a computerised prompt to refer (back) patients with type 2 diabetes
Source: PLoS One. 2018 Dec 5;13(12):e0207653. doi: 10.1371/journal.pone.0207653 (PMC6281259; doi:10.1371/journal.pone.0207653)

**Formulier verzoek advies**

omtrent WMO-plichtigheid van het voorgenomen onderzoek

__________________________________________________________________________

*Gegevens aanvrager*__________________________________________________________________________

Naam Contactpersoon : Drs. M.C.M.Ronda

(procedureel/organisatorisch verantwoordelijk in UMC Utrecht)

Divisie : Julius Centrum

Afdeling : Huisartsgeneeskunde

Huispost : Str. 6.131

Telefoon : 088-7568608

E-mail : m.c.m.ronda@umcutrecht.nl

__________________________________________________________________________

Naam Eindverantwoordelijk onderzoeker : Prof. Dr. G.E.H.M. Rutten

(eindverantwoordelijk onderzoeker in UMC Utrecht, invullen indien dit niet de contactpersoon is)

Divisie : Julius Centrum

Afdeling : Huisartsgeneeskunde

Huispost : Str. 6.131

Telefoon : 088-7568054

E-mail : g.e.h.m.rutten@umcutrecht.nl

Dit formulier is alleen voor UMC Utrecht onderzoekers/werknemers. Studenten dienen hun begeleider als eindverantwoordelijk onderzoeker op te geven.

__________________________________________________________________________

**A. Het onderzoek**

**1. Onderzoektitel:** De juiste patiënt op de juiste plaats.

**2. De vermoedelijke start- en einddatum:**Startdatum 01-08-2013
Einddatum 01-03-2015

**3. Welke vragen tracht uw onderzoek te beantwoorden?**Het doel van dit onderzoek is te bestuderen wat het gevolg is van sturing van zorgverleners binnen een keten-informatie systeem op de behandeling van patiënten met diabetes mellitus type 2.

In de regio Amersfoort wordt alle zorg betreffende 10.000 patiënten met diabetes mellitus type 2 geregeld via de Stichting Diamuraal, waarbij huisartspraktijken in de regio van Amersfoort en het Meander Medisch Centrum zijn aangesloten. De zorgverleners in deze regio werken gezamenlijk in een elektronisch patiënten dossier (EPD) en de patiënten kunnen toegang aanvragen voor hun eigen medische status via internet (genaamd het patiëntenportaal).

Het informatiesysteem van het EPD en het patiëntenportaal is ontworpen door Portavita b.v. Bij Portavita zijn alleen de registratiecodes van de patiënten bekend zoals opgeslagen binnen Diamuraal. Portavita kan deze codes verder niet koppelen aan directe patiëntgegevens, zoals naam, geboortedatum en adres.

Er zijn duidelijke afspraken gemaakt in Diamuraal (“werkprotocol”) met betrekking tot consultatie en (terug)verwijs criteria tussen de 1^e^ en de 2^e^ lijn, welke gebaseerd zijn op de NHG-standaard, Landelijke Transmurale Afspraken en regionale afspraken. Ondanks deze afspraken worden nog niet alle patiënten op de juiste zorgplaats behandeld. Geschat wordt dat bij ongeveer 25% van de patiënten de afspraken in het werkprotocol niet gevolgd worden. De reden hiervoor is onduidelijk en kan zowel met de zorgverlener (bijvoorbeeld door onvoldoende bekendheid met de werkafspraken, niet goed afgestemde werkafspraken of de onbekendheid van de mogelijkheden binnen het EPD) als met de patiënt te maken hebben (niet naar een andere zorgverlener willen, ondanks de richtlijnen).

In dit onderzoek willen we uitzoeken of het EPD ingezet kan worden om de zorg op niveau van de zorgverleners beter te reguleren. Wij willen door middel van een signaal in het EPD de zorgverlener attenderen dat zijn patiënt niet volgens werkprotocol behandeld wordt.

Opzet:

Dit betreft een cluster-randomisatie studie. Huisartspraktijken worden gerandomiseerd naar de interventie- of de controlegroep. Binnen een praktijk doen alle huisartsen mee in dezelfde groep.

Eerst zullen wij (de onderzoeker, MR) beoordelen of de patiënten op de juiste zorgplaats behandeld worden op basis van medische criteria beschreven in het werkprotocol. Bij de groep mensen die op basis van die criteria behandeld worden op de juiste zorgplaats zal verder geen actie ondernomen worden. Bij de groep mensen die op basis van die criteria wel in aanmerking komen voor verandering van zorgplaats zal een vervolg komen.

In dat geval zijn er 4 situaties mogelijk:

1. Patiënten zijn onder behandeling in de 1^e^ lijn, maar zouden volgens de werkafspraken een of meerdere fysieke consulten kunnen vervangen door een zelfcontrole met behulp van het patiëntenportaal;
2. Patiënten zijn onder behandeling in de 1^e^ lijn, maar zouden volgens de werkafspraken een e-consult verdienen met de 2^e^ lijn;
3. Patiënten zijn onder behandeling in de 1^e^ lijn, maar zouden volgens de werkafspraken onder behandeling horen in de 2^e^ lijn;
4. Patiënten zijn onder behandeling in de 2^e^ lijn, maar zouden volgens de werkafspraken terug kunnen naar behandeling door de 1^e^ lijn.

In de *interventiegroep* zal een elektronisch signaal in het EPD gebouwd worden, dat verschijnt bij al hun patiënten dat niet volgens het werkprotocol behandeld wordt (zie een van bovengenoemde 4 situaties). Dit kan dan op het spreekuur met de patiënt besproken en aangepakt worden. Indien de zorgverlener met de patiënt besluit dat de patiënt, ondanks het signaal, niet hoeft te veranderen van primaire behandelaar zal de zorgverlener gevraagd worden in het EPD de reden hiervoor in te vullen. Na 1 jaar evalueren wij wat de gevolgen van dit signaal zijn.

De *controlegroep* zal aan de start van het onderzoek geen signaal ontvangen. Na 1 jaar zullen wij evalueren wat de gevolgen hiervan zijn, met andere woorden, heeft de zorgverlener uit zichzelf de zorgplaats aangepast bij de patiënten bij wie dit nodig was?

Uitkomstmaten van deze studie:

**Primair:**

Aantal extra patiënten dat door het signaal in het EPD verschoven zijn naar de juiste behandelaar ten opzichte van de normale verschuiving, dat wil zeggen de verschuiving die ook zonder signaal plaats heeft gevonden.

**Secundair:**

- Behaalde streefwaarden op gebied van diabetes behandeling (HbA1c, bloeddruk, lipidenspectrum, micro-albuminurie) en correct ingezette benodigde medicatie bij afwijkende streefwaarden (ACE remmer, ARB, statine)

- Patiënt tevredenheid (DTSQ)

- Redenen voor het niet opvolgen van de signalering door de zorgverlener

**B. Proefpersonen**

**1. Aantal proefpersonen:** Stichting Diamuraal bestaat uit 60 huisartspraktijken en het ziekenhuis in Amersfoort. Alle praktijken zullen benaderd worden voor hun meewerking. Gezamenlijk hebben zij rond de 10.000 patiënten met diabetes mellitus type 2. Onze verwachting op basis van een eerder onderzoek is dat ongeveer twee derde van de praktijken mee wil werken (dus een totaal van ongeveer 40 praktijken). Van hun populatie zal naar verwachting twee derde van de patiënten mee willen werken (dus een kleine 5000 patiënten).

**2. Kenmerken van de onderzoekspopulatie:**
De studie-populatie betreft mensen van de leeftijd 18-85 jaar, die in de regio Amersfoort behandeld worden voor diabetes mellitus type 2

**3. Zijn de proefpersonen ook betrokken bij ander al lopend (niet-)WMO-plichtig onderzoek?**Nee.

**C. Methode**

**1. Is er verband met andere studies? Indien ja: METC nummer? WARB nummer?** **Toelichting?** Een deel van deze groep patiënten is eind 2011 benaderd in het kader van het onderzoek: "The use of web-based personal health record in the care of Diabetes Mellitus" (door de METC als niet-WMO-plichtig beoordeeld, METC-protocol nummer 11-296/C, gesubsidieerd door het Diabetes Fonds en door ons uitgevoerd). Dit onderzoek is inmiddels afgelopen. De patiënten hebben voor dat onderzoek eenmalig in eind 2011 een vragenlijst ingevuld.

**2. Geef een beschrijving van de wervingsprocedure van proefpersonen**

Binnen Diamuraal zijn alle zorgverleners en patiënten met diabetes in de regio Amersfoort geregistreerd. Hieruit zullen eerst alle huisartspraktijken worden geworven door middel van een informatiebrief en een informatiebijeenkomst tijdens de huisartsenvergadering Eemland. Op het moment dat praktijken aan geven mee te willen werken aan het onderzoek, zullen alle diabetes type 2 patiënten uit die praktijk een informatiebrief ontvangen met de vraag om toestemming tot het ophalen van medische gegevens uit de database van de huisarts en/of behandelend internist. De internistenpraktijk (vakgroep interne geneeskunde) van Meander Medisch Centrum heeft haar medewerking reeds toegezegd.

**3. Geef een beschrijving van de informed consent procedure**Indien en nadat zorgverleners toestemming hebben gegeven tot deelname aan het onderzoek, zullen patiënten van de betreffende praktijk benaderd worden door middel van een informatiebrief met uitleg over de studie. Ook moeten zij schriftelijk toestemming tot inzage en gebruiken van hun medische gegevens geven middels het meegezonden toestemmingsformulier welke zij kunnen terugzenden door middel van de meegezonden retourenvelop naar de onderzoeker (MR). De onderzoeker zal ook haar contactgegevens in de informatiebrief opnemen, zodat er bij vragen van patiënten met betrekking tot het onderzoek contact opgenomen kan worden met de onderzoeker ter verduidelijking. Na twee weken zal eenmaal een reminderbrief aan patiënten volgen.

**4. Metingen en apparaten: Welke metingen worden er verricht en met welke meetinstrumenten/apparaten?**1) De database van Diamuraal bevat informatie over demografische, antropometrische en diabetes gebonden variabelen, hiermee bepalen we de:

a) indeling op ‘juiste’ behandelaar volgens het Diamuraal werkprotocol .

b) de kwaliteit van diabeteszorg aan de hand van behaalde streefdoelen volgens de

NHG-standaard en LTA richtlijnen (HbA1c, bloeddruk, lipidenspectrum, micro-albuminurie in combinatie met ACE/ARB prescriptie, correct voorgeschreven statine).

(zie bijlage 1 voor alle variabelen)

2) Vragenlijst aan de patiënten: DTSQ vragenlijst (“Diabetes Treatment Satisfaction Questionnaire”). Dit is een gevalideerde vragenlijst die betrekking heeft op de tevredenheid over de diabeteszorg. De vragenlijst bevat acht vragen, geen vragen die diep zullen ingrijpen in de psychische gesteldheid van de patiënten (zie bijlage 2).

3) De zorgverlener zal, indien het signaal niet wordt opgevolgd, direct eenmalig gevraagd worden in het EPD door middel van één meerkeuze vraag aan te geven wat de reden voor het niet opvolgen van het signaal is.

**5. Geef in het geval van meerdere metingen concreet aan wanneer,
welke metingen plaatsvinden (tijdslijn in bijv. uren, dagen, etc.):**

De medische gegevens van patiënten zullen op twee momenten worden opgevraagd. De eerste keer om te kunnen evalueren of de patiënten op de juiste plaats worden behandeld volgens het Diamuraal werkprotocol. De tweede keer aan het eind van het onderzoek om te evalueren of er verandering van behandelaar heeft plaatsgevonden en wat de gevolgen hiervan geweest zijn.

De vragenlijst zal op deze twee momenten ook worden verzonden naar het huisadres van de patiënten, welke ze kunnen terugzenden naar de onderzoeker door middel van een bijgevoegde retourenvelop.

**6. Wat is volgens u de belasting voor de proefpersoon? Gelieve daarbij te onderbouwen in hoeverre de belasting minimaal is.**

Patiënten moeten tweemaal thuis een vragenlijst invullen met acht meerkeuze vragen met een interval van 1 jaar. Indien patiënten op basis van het werkprotocol niet onder behandeling blijken van de juiste zorgverlener zal dit besproken worden tijdens het spreekuur en kan het zo zijn dat patiënten verwezen worden naar de 2^e^ lijn of terugverwezen worden naar de 1^e^ lijn.

**7. Bij afname lichaamsmateriaal: welke hoeveelheid wordt in het kader van het onderzoek afgenomen? Eenmalig of meerdere keren?**

Niet van toepassing.

**8. Is er sprake van speciale bewerking van het lichaamsmateriaal (bijvoorbeeld het kweken van cellijnen?)**

Niet van toepassing.

**9. Zijn er risico’s verbonden aan deelname?**Nee, er zijn geen risico's verbonden aan het onderzoek

**D. Dataverwerking**

**1. Hoe wordt de verkregen data opgeslagen? Tot de persoon herleidbaar of anoniem?**Portavita zal de mogelijkheid van het geven van een signaal moeten inbouwen in het informatie-systeem en activeren voor de interventiegroep. Zij hebben van alle patiënten binnen Diamuraal alleen de registratiecodes, die niet aan verdere persoonsgegevens gekoppeld zijn. Zij kunnen de patiënten niet identificeren, maar wel het betreffende signaal inbouwen bij de juiste registratienummer. De onderzoeker zal aan Portavita aangeven welk signaal aan welke registratiecode gekoppeld moet worden.

De verkregen data zal worden opgeslagen in SPSS onder de registratiecode van de patiënt bij Diamuraal. Deze database met onderzoeksgegevens is in beheer van de onderzoeker op het UMC en zal beveiligd worden. De gegevens zullen alleen herleidbaar zijn voor de onderzoeker, dit is van belang om de gegevens verkregen met de patiënte enquête te koppelen aan de gegevens die uit het data informatiesysteem van Diamuraal verkregen moeten worden. Zij zal de enige zijn met toegang tot deze database.

**E. Gegevens verrichter indien dit niet het UMC Utrecht is**

Niet van toepassing

Datum: 15-01-2013

M.C.M. Ronda

[m.c.m.ronda@umcutrecht.nl](mailto:m.c.m.ronda@umcutrecht.nl)

Utrecht Medisch Centrum

Huispost Stratenum 6.131

Postbus 85500, 3508 GA Utrecht

**Bijlage 1: Variabelen van het onderzoek:**

Gegevens op te vragen uit de database van Diamuraal, met toestemming van de patiënt.

- huidige behandelsituatie (1^e^ lijn zonder consult, 1^e^ lijn met consult afgelopen jaar, 2^e^ lijn,

zelfcontrole; toegang tot EPD)

- type diabetes

- Leeftijd

- BMI (waarden van het afgelopen jaar)

- HbA1c (waarden van het afgelopen jaar) en de eerste glucosewaarde

- Bloeddruk (waarden van het afgelopen jaar)

- Lipidenprofiel: LDL- en HDL-cholesterol, totaal cholesterol en triglyceriden (afgelopen jaar)

- Nierfunctie en complicaties (eGFR, albuminurie, albumine/creatinine ratio)

- Aanwezigheid van overige complicaties (diabetische ulcus, perifeer vaatlijden, myocardaanval,

cerebrovasculair accident, TIA, retinopathie)

- Diabetes medicatie (bloedglucose verlagende medicatie, bloeddruk verlagende medicatie, statine)

**Bijlage 2: Vragenlijst voor de patiënten**

**Tevredenheid met de behandeling (DTSQ)**

| *De volgende vragen gaan over de behandeling van uw diabetes (met insuline, tabletten en/of dieet) en over uw ervaringen gedurende de afgelopen paar weken. Beantwoord elke vraag door voor elke schaal één cijfer aan te kruisen. Hierbij staat de 6 voor het meest positieve antwoord en een 0 voor het meest negatieve.* |
| --- |

**01. Hoe tevreden bent u met uw huidige behandeling?**

□ 6 zeer tevreden

□ 5

□ 4

□ 3

□ 2

□ 1

□ 0 zeer ontevreden

**02. Hoe vaak heeft u de laatste tijd het gevoel gehad dat uw bloedsuikerwaarden te hoog waren?**

□ 6 zeer vaak

□ 5

□ 4

□ 3

□ 2

□ 1

□ 0 helemaal niet

**03. Hoe vaak hebt u de laatste tijd het gevoel gehad dat uw bloedsuikerwaarden te laag waren?**

□ 6 zeer vaak

□ 5

□ 4

□ 3

□ 2

□ 1

□ 0 helemaal niet

**04. Hoe gemakkelijk/handig vindt u uw behandeling de laatste tijd?**

□ 6 zeer gemakkelijk / zeer handig

□ 5

□ 4

□ 3

□ 2

□ 1

□ 0 zeer ongemakkelijk / zeer onhandig

**05. Hoe tevreden bent u met de flexibiliteit (soepelheid) van uw behandeling de laatste tijd?**

□ 6 zeer tevreden

□ 5

□ 4

□ 3

□ 2

□ 1

□ 0 zeer ontevreden

**06. Hoe tevreden bent u over hetgeen u van diabetes begrijpt?**

□ 6 zeer tevreden

□ 5

□ 4

□ 3

□ 2

□ 1

□ 0 zeer ontevreden

**07. Zou u deze vorm van behandeling aanraden aan iemand met uw vorm van diabetes?**

□ 6 ja, ik zou de behandeling zeker aanraden

□ 5

□ 4

□ 3

□ 2

□ 1

□ 0 nee, ik zou de behandeling zeker niet aanraden

**08. Hoe tevreden zou u zijn om uw huidige behandeling voort te zetten?**

□ 6 zeer tevreden

□ 5

□ 4

□ 3

□ 2

□ 1

□ 0 zeer ontevreden

**Bijlage 3: Informatiebrief zorgverleners**

Betreft: informatie en verzoek tot deelname aan onderzoek naar de implementatie van de

diabetes transmurale werkafspraken

Geachte collega,

Regio Eemland kent met Diamuraal een unieke invulling van de diabetes ketenzorg. De zorggroep heeft als doel de diabeteszorg zo efficiënt en patiëntvriendelijk mogelijk in te richten met behoud van kwaliteit en loon naar werken voor de aangesloten zorgverleners.

De zorgverzekeraars stellen ter discussie of de samenwerking binnen Diamuraal wel zo doelmatig is. Onze indicatoren zouden niet excelleren en het aantal patiënten in de 2^e^ lijn zou nog flink verder moeten dalen. Meerdere onder u hebben daarom al hun mening gegeven dat onderzoek het antwoord zou moeten brengen: werken we voldoende volgens de werkafspraken en zo nee, zijn onze patiënten beter af als we daaraan door een signaalfunctie in het KIS (Portavita) extra herinnerd gaan worden? Als we afwijken van de werkafspraken, welke argumenten hebben we daar dan voor? Argumenten die we momenteel niet op gestandaardiseerde wijze in het KIS kunnen vastleggen.

Inmiddels is op verzoek van Diamuraal een onderzoeksplan geformuleerd, waarvoor in een tweetal perioden in 2013 en 2014 uw medewerking wordt gevraagd. De werklast voor de praktijken/instellingen en voor de patiënten zijn daarbij tot een minimum beperkt.

De opzet van het onderzoek

Als eerste willen wij graag in kaart brengen hoe de verdeling van de patiëntenpopulatie over de 1^e^ en 2^e^ lijn is en hoeveel patiënten volgens het zorgprotocol van Diamuraal in aanmerking zouden komen voor ofwel: a) een e-consultatie of fysieke verwijzing van de 1^e^ lijn naar de 2^e^ lijn; b) terugverwijzen van de 2^e^ lijn naar de 1^e^ lijn; c) een vervanging van een kwartaalcontrole in de 1^e^ lijn door een zelfcontrole met behulp van het patiëntenportaal.

Vervolgens zullen wij de deelnemende praktijken door het lot verdelen in 2 groepen: A. een groep waarbij een bericht in het EPD verschijnt, omdat de patiënt mogelijk in aanmerking komt voor een verplaatsing van de zorg (interventiegroep) en B. een groep waarbij dit bericht niet verschijnt en waarbij de zorg zal worden geleverd zoals altijd (controlegroep).

Na 1 jaar gaan wij evalueren wat het effect hiervan is op: daadwerkelijke verschuiving tussen de 1^e^ en 2^e^ lijn, van de 1^e^ lijn naar gecontroleerde zelfzorg en op de kwaliteit van diabeteszorg en de beleving van de patiënten.

Wat betekent deelname aan dit onderzoek voor u zelf:

- Uw patiënten met diabetes mellitus type 2 zullen mede namens u benaderd worden met de

vraag of zij mee willen werken aan dit onderzoek (zie volgende paragraaf).

- Per september 2013 gaan wij het signaal in Portavita introduceren. Indien u geloot heeft voor de

*interventiegroep* kunt u vanaf dat moment , bij de patiënten die mee willen werken aan dit onderzoek, in het EPD een opmerking zien verschijnen waarin aangegeven wordt dat de betreffende patiënt(e) volgens het zorgprotocol in aanmerking zou kunnen komen voor ofwel e-consultatie, ofwel verwijzing naar de internist, ofwel vervanging van een van een kwartaalcontrole door een zelfcontrole. Deze situatie kunt u of uw POH bespreken tijdens het eerstvolgende consult, als de patiënt bij u in de spreekkamer zit. Mocht u besluiten om het aangeboden advies niet op te volgen, dan zal er direct in het EPD de mogelijkheid zijn aan te geven wat hiervoor uw overwegingen zijn.

Indien u geloot heeft voor de *controlegroep*, zal u geen bericht zien verschijnen in het EPD. Aan het einde van de studie (najaar 2014) zult u, bij de patiënten die mee willen werken, alsnog een e-bericht ontvangen in het Portavita-EPD waarin de constatering staat dat zij bij aanvang van de studie volgens het Diamuraal protocol in aanmerking komen voor consultatie of verwijzing.

- De internisten van het Meander Medisch Centrum zullen ook deelnemen aan het

onderzoek, voor hen geldt dat het interventiesignaal zal bestaan uit het verzoek tot terugverwijzen van patiënten naar de 1^e^ lijn.

- U zult een vergoeding ontvangen ter compensatie van de tijd besteed door u of uw

praktijkmedewerkers om deze gezamenlijke missie van huisartsen, Meander Medisch Centrum en Diamuraal te volbrengen.

**Notabene:** wij verzoeken u extra goed te letten op het invullen van de nieuwe medicatie-vragen, die bij de komende release aan de jaarcontroles en kleine controles zijn toegevoegd, omdat gegevens zoals gebruik van RAS-blokkerende middelen (ACE-remmers, A2-antagonisten, renine-blockers) onderdeel gaan uitmaken van de zorgkwaliteitsscore.

Wat betekent deelname aan dit onderzoek voor uw patiënt:

Nadat u toestemming heeft gegeven tot deelname aan dit onderzoek, zullen wij vanuit Diamuraal, mede uit uw naam, uw patiënten benaderen met verzoek tot deelname. Achtergrond en opzet van de studie zullen worden uitgelegd.

Wij vragen mede namens u aan uw patiënten:

- Toestemming tot het gebruiken van de persoonlijke, diabeteszorg gerelateerde

gegevens uit Portavita voor de onderzoeksdoeleinden.

- Het tweemaal invullen van een vragenlijst, die zij aan het begin en aan het eind van de studie

thuisgestuurd zullen krijgen en aan ons kunnen retourneren.

- Indien u als huisarts in de interventiegroep zit zal bij de patiënten die toegang hebben tot het patientenportaal een voorzichtig geformuleerde signalering verschijnen dat er op basis van de huidige medische gegevens mogelijk sprake zou kunnen zijn van een aanpassing in “zorgplaats”. Dit maakt het bespreken hiervan tijdens het consult makkelijker voor u.

Wij hopen met de uitkomsten van dit onderzoek meer zicht te krijgen of de patiënt de juiste zorg op de juiste plaats ontvangt, waarna verwijs- en consultatiecriteria binnen Diamuraal indien nodig aangepast kunnen worden. Voorts zal de toegevoegde waarde van een automatische signalering binnen dit proces geëvalueerd kunnen worden. Wij willen benadrukken dat dit onderzoek bedoeld is om bestaande afspraken waar mogelijk te verbeteren en te nuanceren en niet om u als zorgverlener te controleren of te beoordelen.

Voor suggesties aangaande de informatieverstrekking aan uw patiënten (zie bijlage 2) staan wij open. Alvast veel dank voor uw medewerking!

Met vriendelijke groet,

Maaike Ronda

Onderzoeker te UMCU en huisarts in opleiding

Tel nr: 088-7568608

m.c.m.ronda@umcutrecht.nl

Mede namens,

Leo Boom, medisch directeur Diamuraal en huisarts

Lioe-Ting Dijkhorst, senioronderzoeker en internist Meander Medisch Centrum

Guy Rutten, hoogleraar diabetologie UMCU en huisarts

Bijlagen:

1. Gegevens op te halen uit Portavita
2. Voorbeeld informatiebrief aan patiënten.
3. Toestemmingsformulier, hierin staan kort de taken van de huisarts waaraan hij of zij zich committeert.

**Toestemmingsformulier**

**“De juiste patiënt op de juiste plaats”**

METC-protocolnummer:

Ondergetekende verklaart dat hij/zij zich inschrijft voor de studie “De juiste patiënt op de juiste plaats” en onderschrijft de onderstaande voorwaarden en afspraken:

- Ik heb de informatiebrief gelezen en begrepen.

- Ik wil meewerken aan dit onderzoek zoals beschreven in de informatiebrief.

- Verder zal ik ervoor zorgdragen dat:

a. De gegevens die nodig zijn ter beoordeling van zorgplaats zoals genoemd in bijlage 1 zo

volledig mogelijk zijn ingevuld in Portavita.

b. Mijn patiënten uit mijn naam benaderd mogen worden voor deelname aan dit onderzoek.

c. Op het moment dat ik een adviessignaal in het EPD niet opvolg, zal ik in het EPD aangeven

wat de reden hiervoor is. Dit zal in de interventiegroep direct na start van de studie zijn, in de controlegroep zal dat na afloop van de studie zijn.

Invullen wat van toepassing is:

**Ik wil meedoen aan dit onderzoek en geef toestemming tot bovenstaande.**

Naam:

Handtekening: Datum : __ / __ / __

**Ik wil niet meedoen aan dit onderzoek en geef geen toestemming tot bovenstaande.**

Naam:

Handtekening: Datum : __ / __ / __

-----------------------------------------------------------------------------------------------------------------

Ik verklaar hierbij dat ik bovenstaande persoon volledig heb geïnformeerd over het genoemde onderzoek door middel van bijgevoegde informatiebrief met bijlagen.

Naam onderzoeker: Maaike Ronda

Datum: 15 / 01 / 2013


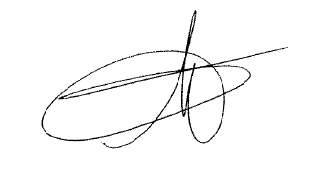


**Bijlage 4: Informatiebrief patiënten**

**Informatiebrief: De Juiste Patiënt op de Juiste Plaats**

Geachte heer/mevrouw,

Hierbij vragen wij u vriendelijk mee te doen met het onderzoek “ de juiste patiënt op de juiste plaats”. Voordat u deze beslissing neemt, is het belangrijk meer te weten over het onderzoek. Daarvoor is deze informatiebrief bedoeld. Lees deze informatiebrief rustig door en bespreek deze indien u dit wenst met uw partner, vrienden en/of familie. Indien u na het lezen van deze informatie nog vragen heeft kunt u contact opnemen met de onderzoeker. Op bladzijde 5 vindt u haar contactgegevens.

**Wat is het doel van het onderzoek?**

Op dit moment zijn wij bezig met een onderzoek naar de zorg die rondom de behandeling van diabetes plaats vindt. Wij willen samen met uw zorgverlener onderzoeken of de afspraken die rondom de diabeteszorg in de regio gemaakt zijn tussen de huisartspraktijken en het ziekenhuis voldoende zijn, dan wel verbeterd kunnen worden. Op dit moment zijn deze afspraken vastgelegd in een zogenaamd “werkprotocol”. Deze afspraken gaan onder andere in op de taakverdeling van de huisarts en de specialist in het ziekenhuis (internist). Door het daar waar nodig optimaliseren van deze taakverdeling willen wij de diabetes zorg verbeteren. De reden dat wij u benaderen is omdat wij de afspraken willen evalueren aan de hand van de gegevens omtrent uw gezondheid, hiervoor is uw toestemming nodig.

**Hoe wordt het onderzoek uitgevoerd?**

Zoals u misschien weet is uw arts aangesloten bij Stichting Diamuraal. Dit is een regionaal samenwerkingsverband dat er voor zorgt dat zorg tussen de eerste lijn (huisartsen) en tweede lijn (internisten) goed op elkaar afgestemd is. Al uw gegevens zijn opgeslagen in een elektronisch patiënten dossier waartoe alleen uw zorgverlener toegang toe heeft. Tijdens het consult worden uw gegevens over de diabetes hierin genoteerd. Velen van u (25% van de patiënten behandeld binnen Stichting Diamuraal) hebben op eigen verzoek toegang tot het Digitaal Logboek, waarmee ze thuis ook hun elektronisch medisch dossier kunnen inzien en gebruiken.

Wat wij gaan doen tijdens dit onderzoek is in dit elektronische dossier een extra signaal inbouwen. Op het moment dat uw zorgverlener uw dossier opent terwijl u bij hem/haar op het spreekuur bent, zal hij/zij meteen kunnen zien hoe het met uw bloedwaarden staat en welke van die waarden eventueel extra aandacht nodig hebben. Hiervoor hebben wij een aantal van uw gegevens nodig, zoals de hoogte van uw HbA1c en uw bloeddruk (zie bijlage 1). Aan de hand van deze informatie zullen wij nagaan of u conform het werkprotocol onder behandeling bent bij de voor uw situatie meest aangewezen zorgverlener.

Hierbij zijn de volgende situaties mogelijk:

a) U bent onder behandeling bij uw huisarts of internist en gegevens wijzen erop dat dit voor

u de meest aangewezen zorgverlener is.

b) U bent onder behandeling bij uw huisarts en uw waarden zijn zodanig goed dat u minder vaak (bijvoorbeeld 2x in plaats van 4x per jaar) op het spreekuur hoeft te komen, mits u

uw Digitaal Logboek gaat gebruiken.

c) U bent nu onder behandeling bij de huisarts maar als gevolg van een of meerdere van uw

waarden kan het wenselijk zijn dat er overleg plaatsvindt, bijvoorbeeld eenmalig, tussen uw huisarts en de internist (u blijft onder behandeling van uw huisarts). Het zou ook kunnen betekenen dat u bij de internist op het spreekuur zou moeten komen.

d) U bent nu onder behandeling bij de internist en uw waarden zijn inmiddels weer zodanig

dat u niet langer onder behandeling hoeft te blijven bij de specialist en terug kunt naar

uw huisarts voor de verdere behandeling.

In situatie [a] houdt het onderzoek voor u verder op en hoeft u niets meer te doen. U krijgt en houdt de zorg zoals u gewend bent.

Op het moment dat bij u situatie b, c of d van toepassing is verschijnt er een signaal in het elektronische medische dossier. De eerst volgende keer dat u op het spreekuur komt ziet uw zorgverlener dit en zal dit dan met u bespreken. **Daarna beslist u samen hoe en waar u de diabetesbehandeling verder wilt krijgen.**

De verwachting is dat dit soort automatische signalen in de toekomst steeds meer ingebouwd zullen worden om zorgverlening te ondersteunen. Om goed te kunnen beoordelen of patiënten en zorgverleners voordeel hebben van een dergelijke signalering, willen wij de zorgverleners verdelen in 2 groepen.

- Bij groep [1] verschijnt het signaal direct bij het openen van uw dossier. Uw arts zal dit

dan meteen met u bespreken en samen met u besluiten of het signaal opgevolgd zal

worden. Na een jaar zullen wij gaan beoordelen of uw zorgverlener het advies van het

signaal als nuttig heeft ervaren, of u door het signaal bij een andere zorgverlener op het

spreekuur bent gekomenen en of er betere bloedwaarden gerealiseerd zijn. Uiteraard

willen wij van u weten of uzelf tevreden bent.

- Bij groep [2] zal in het eerste jaar geen signaal gegeven worden. U krijgt gewoon de zorg

die u gewend bent. Na een jaar zal alsnog het signaal verschijnen. Dat zal op dat moment

met u besproken worden. Dan kan samen met u besloten worden of het signaal (alsnog) opgevolgd zal worden of niet. Wij willen ook onderzoeken of u – zonder dit signaal –

inmiddels bij een andere zorgverlener op het spreekuur bent geweest, of uw bloedwaarden verbeterd zijn en of u zelf tevreden bent.

Met behulp van dit onderzoek hopen wij te bereiken dat door middel van een automatische signaalfunctie de zorg verbeterd kan worden door de zorg zo efficiënt mogelijk in te richten.

**Wat wordt er van u verwacht?**

Voor dit onderzoek hebben wij twee keer uw gegevens nodig. De eerste keer dat de gegevens ingezien worden is aan het begin van het onderzoek. Dit is om te beoordelen onder welke van de afspraken van het “werkprotocol” u valt. De tweede keer is na een jaar, om te beoordelen of uw behandeling veranderd is van plaats en om in te zien wat het gevolg hiervan is (heeft het geholpen om uw waarden te verbeteren?).

Deze gegevens zullen anoniem worden verwerkt en opgeslagen. Alleen de onderzoeker heeft toegang tot deze gegevens. Hiervoor hebben we uw toestemming nodig (zie bijlage 2). Van het eenmalig aan het begin en eenmalig aan het eind van het onderzoek inzien van de gegevens zelf merkt u verder niets.

Indien u behoort tot groep [2] en een of meerdere van uw waardes aanleiding geven voor een signaal zult u dit merken doordat uw zorgverlener dit met u zal bespreken op het spreekuur. Indien u al thuis gebruik maakt van het Digitale Logboek, dan zult u dat ook zelf kunnen zien.

Tot slot willen wij aan iedereen vragen om twee keer, één keer aan het begin van het onderzoek en één keer aan het eind (dat wil zeggen na 1 jaar) een vragenlijst in te vullen met vragen of u tevreden bent over de zorg die u krijgt. Deze vragenlijst bevat 8 korte vragen en kunt u bij deze brief vinden (zie bijlage 3).

**Wat zijn mogelijke voor- en nadelen van deelname aan dit onderzoek?**

Dit onderzoek zal nuttige gegevens opleveren voor het verbeteren van de bestaande afspraken over de diabeteszorg. Voor uzelf kan het zo zijn dat door dit onderzoek blijkt dat u in overleg met uw zorgverlener naar een andere zorgverlener verwezen wordt. Op de lange termijn helpt dit onderzoek bij het op de juiste manier ontwerpen/verbeteren van uw elektronisch patiëntendossier voor diabetes met automatische behandeladviezen om u en uw zorgverlener te ondersteunen in de diabeteszorg.

**Wat gebeurt er als u niet wenst deel te nemen aan dit onderzoek?**

Dit onderzoek is geheel vrijwillig. Als u besluit niet mee te doen dan kunt u dat aangeven op het bijgevoegde formulier genaamd: “toestemmingsformulier”. Indien u hierop aangeeft dat u niet wenst mee te doen met het onderzoek, dan geeft u daarmee te kennen geen toestemming te geven en zullen wij u niet opnieuw benaderen. Indien u niet meedoet met het onderzoek heeft dat geen gevolgen voor uw behandeling. U zult dan gewoon uw behandeling voor de diabetes krijgen die u anders ook zou krijgen.

**Wat gebeurt er met uw gegevens?**

Uw gegevens zullen geanonimiseerd onder een code worden opgeslagen. Alleen de onderzoeker kent deze code. Uw gegevens worden alleen gebruikt om te beoordelen of u de zorg op de juiste plaats krijgt (en waarom wel of niet), hoe die zorg uitpakt (diabetesmedicijnen en waarden) en hoe tevreden u hierover bent. Verder zullen uw gegevens nergens anders voor gebruikt worden en heeft niemand anders toegang tot deze gegevens. U heeft ten alle tijden recht om te verzoeken uw gegevens te laten verwijderen.

**Wordt uw huisarts en/of behandelend specialist geïnformeerd bij deelname?**

Uw huisarts zal niet apart op de hoogte worden gesteld of u wel of niet mee werkt aan dit onderzoek. Indirect zal uw zorgverlener, indien deze behoort tot de groep waarbij het signaal wel aan staat en een signaal gegeven wordt er van op de hoogte zijn dat u meedoet.

**Welke medisch-ethische toetsingscommissie heeft dit onderzoek goedgekeurd?**

Toetsingscommissie heeft dit onderzoek goedgekeurd onder nummer [X].

**Wilt u verder nog iets weten?**

Heeft u verder nog vragen, dan kunt u contact opnemen met de onderzoeker. Zij is bereikbaar onder telefoonnummer 088-7568608 en via e-mail: [m.c.m.ronda@umcutrecht.nl](mailto:m.c.m.ronda@umcutrecht.nl)

**Als u wilt meewerken aan dit onderzoek dan vragen wij u vriendelijk het bijgesloten toestemmingsformulier en vragenlijst in te vullen en aan ons terug te sturen met de antwoordenvelop.**

Met vriendelijke groet,

Maaike Ronda,

huisarts in opleiding en onderzoeker te UMCU

Mede namens,

… (betreffende huisarts of internisten)

En namens het onderzoeksteam:

Drs. L.A. Boom, medisch directeur Stichting Diamuraal en huisarts

Dr. L.T. Dijkhorst-Oei, internist in het Meander Medisch Centrum

Dr. G.E.H.M. Rutten, huisarts en hoogleraar diabetologie te UMCU

**Bijlagen:**

1. De gegevens die wij over u willen opvragen bij Stichting Diamuraal.

2. Toestemmingsformulier.

3. Vragenlijst over uw tevredenheid over de zorg.

**Toestemmingsformulier**

**“De juiste patiënt op de juiste plaats”**

Ondergetekende verklaart dat hij/zij zich inschrijft voor de studie “De juiste patiënt op de juiste plaats” en onderschrijft de onderstaande voorwaarden en afspraken:

- Ik heb de informatiebrief gelezen en begrepen.

- Ik wil meewerken aan dit onderzoek zoals beschreven in de informatiebrief.

Invullen wat van toepassing is:

**□ Ik wil meedoen aan dit onderzoek en geef toestemming tot bovenstaande.**

**□ Ik wil niet meedoen aan dit onderzoek en geef geen toestemming tot bovenstaande.**

Naam: de heer / mevrouw

Geboortedatum:

Handtekening: Datum : __ / __ / __

----------------------------------------------------------------------------------------------------------------

Ik verklaar hierbij dat ik bovenstaande persoon volledig heb geïnformeerd over het genoemde onderzoek door middel van bijgevoegde informatiebrief met bijlagen.

Naam onderzoeker: Maaike Ronda

Datum: 15 / 01 / 2013


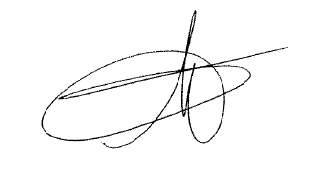

Supplement: S2 File — Protocol submitted to Medical Ethics Committee of the University of Utrecht for assessment if participants are subjected to the Medical Research Involving Human Subject Act (WMO). In Dutch. (DOCX) [file pone.0207653.s004.docx]
