# Supplementary material for: Cluster randomised trial on the effectiveness of a computerised prompt to refer (back) patients with type 2 diabetes
Source: PLoS One. 2018 Dec 5;13(12):e0207653. doi: 10.1371/journal.pone.0207653 (PMC6281259; doi:10.1371/journal.pone.0207653)
Supplement: S3 File — (DOCX) [file pone.0207653.s005.docx]

**Study Form**

**A. Project**

**1. Project title:** The right patient in the correct setting.

**2. The planned start- and end date:**Start date 01-08-2013
End date 01-03-2015

**3. What are the questions you want answered with your project?**The purpose of this project is to study the effect of supporting care providers in a care group setting with the treatment with patients with diabetes mellius type 2.

In the region of Amersfoort, all the care of 10.000 patients with type 2 diabetes mellitus is provided by Diamuraal, a care group of reginal primary care practices and the Meander Medical Centre hospital. All health care providers work with the same electronic medical record (EMR) and patients can request a login to a patient web portal that gives them access to their entire personal EMR. The EMR system and patient web portal are designed by Portavita. Portavita solely has access to the registration codes of Diamuraal patients, and is not able to match this with direct patient information, e.g. name, date of birth and address.

There are clear agreements within Diamuraal (“workprotocol”) regarding criteria for consultation, referral and back referral between primary care practices and the hospital. These are based on the national clinical guideline on patients with type 2 diabetes in primary care (NHG-standaard), the national agreement between primary care practitioners and internists on type 2 diabetes mellitus (Landelijke Transmurale Afspraken, “LTA”) and regional agreements. Despite these agreements, not all patients are treated in the correct setting, probably in 25% of patients. Reason for this is unclear and is probably due to both physician factors (e.g. unfamiliarity with the workprotocol or unfamiliarity with EMR functionalities) and patient factors (e.g. preference for their personal health care provider).

With this study we aim to investigate if the EMR can be used to support care providers in improving allocation of care of patients with type 2 diabetes. We will add an automatic signal in the EMR that will alert the care provider when a patients is not treated according protocol.

Methods:

This is a cluster-randomized trial. Medical practices will be randomized in intervention- and control group. Per practice all physicians will participate within the same group. First we (the researcher, MR) will evaluate if a patient is treated in the correct setting based on medical guidelines. If a patient is treated in the correct setting no further action will follow. If a patient is not treated in the correct setting, they are eligible for this study. If that is the case there are four possible situations:

1. Patients are treated in the primary care, but are eligible for substituting a regular consult into a self-control with help from the patient portal.
2. Patients are and remain treated in the primary care but should receive an e-consult with an internist.
3. Patients are treated in the primary care but should be referred to hospital based internist.
4. Patients are treated in the hospital (internist) but should be referred back to primary care.

In the *intervention group* an automatic electronic signal will pop-up in the EMR, showing that the patient is not treated in the correct setting (one of the above mentioned 4 situations). A physician then can discuss this during consult with the patient and correct this situation. If the physician and patient together decide this is not necessary, we will ask the physician to explain the reasons for not following the guideline, which can be noted in the EMR. After one year we will evaluate the effect of this advice signal.

The *control group* will not receive an automatic electronic signal. After one year we will evaluate the effect; has the physician corrected the setting without the help/reminder of the electronic signal?

Outcomes:

**Primary:**

The number of participants with change in allocation of care after intervention

**Secondary:**

- Change in quality of diabetes care (QuED score) and reached targets (HbA1c, blood pressure, lipids, micro-albuminuria, medication regimen)

- Change in patient treatment satisfaction questionnaire (DTSQ)

- Assembly of reasons by health care provider for not following the guidelines

**B. Participants**

**1. Number of participants:** Diamuraal consists of 60 primary care practices and one hospital (Amersfoort). All practices and physicians will be invited to participate. Together they treat over 10.000 patients with type 2 diabetes mellitus. We expect, based on a previous study, that two-third is willing to participate (approximately 40 practices). We also expect that two-third of their patients is willing to participate (approximately 5000 patients).

**2. Eligibility criteria of participants:**
The study-population are people aged 18-85 years, treated for type 2 diabetes mellitus in the region of Amersfoort.

**3. Are the participants already included in another study?**No.

**C. Method**

**1. Is there a connection with other studies? If yes: METC number? WARB number?** **Explain.** Some of these patients were invited to participate in another study in 2011: "The use of web-based personal health record in the care of Diabetes Mellitus" (METC-protocol number 11-296/C, funded by the Diabetes Fund and executed by our research team). This study is finished. For this study the patients had to fill in one questionnaire in 2011.

**2. Give a description of invitation of participants.**

In Diamuraal all health care providers and patients with type 2 diabetes mellitus n the region of Amersfoort are registered. First we will invite the practices, using an informational letter and giving information during a meeting of the primary care physicians Eemland. If practices agree to participate, we will send all patients with type 2 diabetes within that practice an informational letter and ask permission to collect medical data from the electronic medical record database. The internists of the Meander Medical Center Hospital already agreed to participate.

**3. Describe the informed consent procedure.**If and when the physicians agreed to participate, patients will be informed by means of an informational letter explaining the study. They have to give written consent for collecting and using medical data, the consent form can be returned in a provided postal envelop and is sent to the researcher (MR). The researcher will provide contact information in the informational letter, so patients can contact the researcher to answer remaining questions. After 2 weeks an reminder letter will be sent to patients.

**4. Measures: Which measures will be performed and with what measuments/equipments?**1) The central database of Diamuraal contains information about demographical, antropomic and diabetes related variabelen, we use this to evaluate:

a) assessment of ‘correct’ setting according to Diamuraal workprotcol,

b) quality of diabetes care evaluated by reached treatment targets according to

NHG-standaard and LTA guidelines (HbA1c, blood pressure, lipids, micro-albuminuria in combination with ACE/ARB prescription, correct prescribed statin).

(see appendix 1 for all variables)

2) Questionnaires to patients: DTSQ questionnaire (“Diabetes Treatment Satisfaction Questionnaire”). This is a validated questionnaire on patient satisfaction regarding diabetes care. It contains eight questions (see appendix 2).

3) For the physician there is one multiple question in the EMR that needs to be filled in when it is decided not to follow the guideline to change the treatment setting (regarding the reasons for this).

**5. In case of multiple measurements, state the time and which measurements will be performed.**

The medical data will be collected at two moments. The first time to evaluate which patients are not treated correctly according to workprotocol. The second time data is collected is at the end of the study to evaluate the effect of the intervention.

The questionnaire to patients will be sent at these two moments as well, they can return this with the provided postal envelop.

**6. State the toll for the participants.**

Patients have to fill in a questionnaire twice (8 questions), at home with a one-year interval. If after evaluation, a patient is not treated in the correct setting this will be discussed during consultation with the physician and hence it is possible patients will change treatment setting (referral to internist or back-referral to primary care).

**7. In case of collecting biomedical material: how much will be collected? Once or multiple times?**

Not applicable.

**8. Is there special processing of the biomedical material (e.g. cultivating cell cultures?)**

Not applicable.

**9. Are there risks in participating this study?**No there are no risks.

**D. Processing of data**

**1. How will the collected data be stored? Recognizable or anonymous?**Portavita will built the signal in the informational-system and activate this for the intervention group. They only have the patient registration codes from Diamuraal, no other personal information. They are not able to identify patients, but are able to send the signal to the correct registration code. The researcher will give Portavita the registration code of patients that need a signal.

The data will be stored in SPSS with the Diamuraal registration code. This database is in control of the researcher at the University Medical Center Utrecht and will be password protected. The data only is identifiable for the researcher, this is necessary to connect the data from the EMR with the patient satisfaction questionnaires. She will be the only one with access to this database.

**E. Contact information in case the reseachre is not connected to the UMC Utrecht**

Not applicable.

Datum: 15-01-2013

M.C.M. Ronda

[m.c.m.ronda@umcutrecht.nl](mailto:m.c.m.ronda@umcutrecht.nl)

Utrecht Medisch Centrum

Huispost Stratenum 6.131

Postbus 85500, 3508 GA Utrecht

**Appendix 1:** variables retrieved from the central database

**Appendix 2:** DTSQ

**Appendix 3:** Informational letter to physicians (including an informed consent form)

**Appendix 4:** Informational letter to patients (including an informed consent form)

**Appendix 1: Variables retrieved from the electronic medical record database**

- current treatment setting (primary care without consult, primary care with consult within the

previous year, hospital care, selfcare; access to EMR/patient portal)

- type of diabetes

- age

- body mass index (most recent value in the previous year)

- HbA1c (most recent value in the previous year) and the first glucose value

- Blood pressure (most recent value in the previous year)

- Lipids: LDL- and HDL-cholesterol, total cholesterol and triglycerides (most recent value in the

previous year)

- Kidney-function and complications (eGFR, albuminuria, albumin/creatinine ratio)

- Presence of other complications (diabetic ulcer, peripheral arterial disease, myocardial infarction,

cerebrovascular accident, transient ischemic accident, retinopathy)

- Medication (blood glucose lowering medication, blood pressure lowering medication, statin)
